# Evaluating precision medicine tools in cystic fibrosis for racial and ethnic fairness

**DOI:** 10.1017/cts.2024.532

**Published:** 2024-05-07

**Authors:** Stephen P. Colegate, Anushka Palipana, Emrah Gecili, Rhonda D. Szczesniak, Cole Brokamp

**Affiliations:** 1 Division of Biostatistics and Epidemiology, Cincinnati Children’s Hospital Medical Center, Cincinnati, OH, USA; 2 School of Nursing, Duke University, Durham, NC, USA; 3 Department of Pediatrics, University of Cincinnati, Cincinnati, OH, USA

**Keywords:** Cystic fibrosis, group fairness, lung function, medical monitoring, precision medicine, pulmonary function tests

## Abstract

**Introduction:**

Patients with cystic fibrosis (CF) experience frequent episodes of acute decline in lung function called pulmonary exacerbations (PEx). An existing clinical and place-based precision medicine algorithm that accurately predicts PEx could include racial and ethnic biases in clinical and geospatial training data, leading to unintentional exacerbation of health inequities.

**Methods:**

We estimated receiver operating characteristic curves based on predictions from a nonstationary Gaussian stochastic process model for PEx within 3, 6, and 12 months among 26,392 individuals aged 6 years and above (2003–2017) from the US CF Foundation Patient Registry. We screened predictors to identify reasons for discriminatory model performance.

**Results:**

The precision medicine algorithm performed worse predicting a PEx among Black patients when compared with White patients or to patients of another race for all three prediction horizons. There was little to no difference in prediction accuracies among Hispanic and non-Hispanic patients for the same prediction horizons. Differences in F508del, smoking households, secondhand smoke exposure, primary and secondary road densities, distance and drive time to the CF center, and average number of clinical evaluations were key factors associated with race.

**Conclusions:**

Racial differences in prediction accuracies from our PEx precision medicine algorithm exist. Misclassification of future PEx was attributable to several underlying factors that correspond to race: CF mutation, location where the patient lives, and clinical awareness. Associations of our proxies with race for CF-related health outcomes can lead to systemic racism in data collection and in prediction accuracies from precision medicine algorithms constructed from it.

## Introduction

Cystic fibrosis (CF) is a disease that causes the production of abnormally thick secreted fluids [1–3], especially inside the lungs and the pancreas [1–7]. As a result, CF lung disease progression is marked by recurring rapid declines in lung function in the form of acute respiratory events, clinically referred to as pulmonary exacerbation (PEx) events [8–10]. Symptoms include coughing, sputum production, wheezing, chest tightness, difficulty breathing or shortness of breath, and fever [11]. Precision medicine algorithms that predict these attenuated declines in CF have been developed in recent years [12,13]. While many of these algorithms entail different approaches, their primary purpose is to direct care and resources to high-need CF patients at the right time [14]. Development and targeted use of advanced personalized treatments such as ivacaftor and lumacaftor are highlights of the CF community embracing precision medicine [15]. Precision medicine, however, permits discriminatory and harmful impacts of structural racism that could potentially impact groups that have been historically marginalized [16]. Racial bias can be introduced in building and analyzing datasets, but it can also be the result of precision medicine research [14].

Sparking much of the latest interest in PEx prediction was the CF Foundation Learning Network’s adaptation of a data-driven definition of PEx. They considered changes in lung function, measured as a forced expiratory volume in 1 s (FEV_1_) of % predicted, relative to the baseline to identify PEx. The definition is known as the FEV_1_-indicated exacerbation signal (FIES) and is applied over time for each individual patient [17]. We have developed a nonstationary Gaussian stochastic process model to predict PEx using demographic, encounter-level, and hospitalization data from the US CF Foundation Patient Registry (CFF-PR) [13]. This precision medicine algorithm incorporates clinical measures and has been expanded to include place-based measures to forecast FIES risk [18] including traffic (on the basis of primary road density and secondary road density of the ZIP code tabulation area), community material deprivation, and greenspace. The algorithm then determines the probability a CF patient will experience a future PEx event from the date of an encounter by forecasting FIES risk. The model has been shown to accurately predict rapid lung function declines up to two years from a clinical evaluation (median area under the receiver operating characteristic [ROC] curve 0.817, 95% confidence interval [CI]: 0.814, 0.822), serving as a useful clinical tool to identify for whom and when a FIES-defined PEx event is imminent [19]. Earlier prediction and identification of a PEx event allow for earlier preventative interventions; therefore, the accuracy of any precision health algorithm will influence an individual’s morbidity and mortality.

Concerns around racial equity in many precision health algorithms have been introduced [20,21] because they include race as a predictor [22], but racial inequality is also present in real-world precision health algorithms that do not explicitly use race as a predictor [23]. Group-level fairness is defined as the desired state of achieving similar model performance across subpopulations partitioned by protected attributes, such as race and ethnicity [24]. Most precision medicine research today, however, does not incorporate group fairness adequately into the statistical evaluation process and may not even consider group bias for aspects such as variable measurement and design selection [25–27]. Rather, researchers focus on the accuracy and interpretation of the algorithm being developed and are only evaluated based on individual fairness – similar individuals within a population being treated similarly [28]. Even though symptom severity and frequency vary between individuals, precision medicine algorithms commonly use covariates associated with race and ethnicity [29] that could induce differences in PEx prediction accuracies between racial and ethnic groups. Using predictors associated with race or ethnicity does not necessarily imply predictions will be unfair, but we do not wish to see racial and ethnic differences in accuracies in PEx prediction from our precision medicine algorithm. We screened predictors from our PEx precision medicine algorithm to determine if they are correlated with race or ethnicity, as this is most likely how racial and ethnic differences are incorporated in prediction accuracies.

## Methods

The CFF-PR collects information on CF patient encounters who receive care in CF Foundation-accredited care centers [30]. Race and ethnicity information was collected using categories (White, Black or African American, Other, Asian, Native Hawaiian or other Pacific Islander, American Indian or Alaskan native) defined by the CF Foundation based on patient-level clinical records from each site [30]. We then further categorized the patient’s race as either “White” if they identified as only White, “Black” if they identified as Black, and “Other” if any other race (besides White or Black) was selected. We defined a patient’s ethnicity whether they self-identified as either Hispanic or non-Hispanic. The CFF-PR cohort was primarily composed of White patients (White: *n* = 24,490 [92.8%], Black: *n* = 1,172 [4.4%], Other: *n* = 730 [2.8%]) and non-Hispanic patients (non-Hispanic: *n* = 23,392 [88.6%], Hispanic: *n* = 2,045 [7.7%]) with CF. There were 955 patients (3.6%) who did not report their ethnicity, due to a combination of patients refusing to report and healthcare centers not collecting this information. We did not consider patients with unknown ethnicity since the rate of missingness differs within each racial group (White: *n* = 691 [2.6%, Other: *n* = 206 [2.8%], Black: *n* = 58 [0.2%]).

The precision medicine algorithm models personalized thresholds of rapid lung function decline [13]. The algorithm is a non-stationary stochastic process model comprised of fixed effects (age, F508del, birth cohort, FEV_1_ at baseline, enzyme use, *Pseudomonas aeruginosa*, methicillin-resistant *Staphylococcus aureus*, Medicaid insurance use, CF-related diabetes mellitus, outpatient visits in last year, acute exacerbations in last year), between-patient heterogeneity, and a continuous-time integrated Brownian motion process to determine hyperlocal, dynamic predictive probabilities in lung function (FEV_1_) measurement. The algorithm was applied to data from 30,879 US CFF-PR patients, and the median (95% CI) area under the ROC curve estimates was 0.817 (0.814, 0.822). While the precision medicine algorithm has reasonable accuracy in personalized rapid lung function decline predictions, the algorithm has not been evaluated for group fairness.

We analyzed each predictor and outcome to determine if associations with race and ethnicity could be responsible for any model unfairness. Predictors considered include gender (male, female), F508del mutation (homozygous, heterozygous, neither/unknown), insurance payor status (private or non-private), smoking status (smoker, nonsmoker), smoking household (yes, no), secondhand smoke (yes, no), primary road density and secondary road density as a proxy for traffic exposure (total length of all roads in meters in the ZIP Code Tabulation Area [ZCTA] divided by the total area in square meters of the ZCTA), a community material deprivation index [31] (a census tract-level deprivation index based on five different census tract-level variables related to material deprivation, derived from the 2015 5-year American Community Survey), fraction of surrounding land characterized as greenspace (using the National Land Cover Database), straight-line distance (in meters) and drive time (in 5-minute intervals) to the healthcare center, baseline age (patient’s age at first encounter), number of encounter visits, number of PEx events, and the amount of time since baseline age at encounter.

Rapid lung function decline is defined by a decrease in FEV_1_ of more than 10% predicted from the maximum observed FEV_1_ within the past 12 months [32,33]. We identified a PEx using predictions of rapid lung function decline based on FIES [34–36]. FIES is determined by first defining the patient’s baseline FEV_1_ as the average of the highest two FEV_1_ measurements in the past 12 months when not under intravenous antibiotic treatment [37]. If only one valid FEV_1_ was available, it was used as the baseline. A FIES-defined PEx event occurs when the FEV_1_% predicted declines at least 10% or more [34]. The FIES definition excludes any lung function measurements within 28 days of a previous PEx to ensure accurate patient baseline FEV_1_. We calculated prediction accuracy by comparing the PEx probability from the precision medicine algorithm with whether a PEx eventually occurred for 3-, 6-, and 12-month prediction horizons.

A ROC curve [38] was used to determine the optimal cutoff probability for a PEx occurrence. Group-specific ROC curves were then implemented using this optimal cutoff probability to achieve group-specific sensitivity and specificity. ROC curves were formulated by contrasting a patient’s PEx probability from the precision medicine algorithm to actual PEx outcomes for the prediction horizon at each clinical visit. Actual PEx outcomes were determined by whether the patient was clinically evaluated and confirmed to have at least one PEx within the prediction horizon. The area under the ROC curve (AUC), sensitivity, and specificity were calculated overall and for each group-specific subpopulation using Youden’s J statistic [39]. The AUC was used as a benchmark for the PEx precision medicine algorithm performance [40]. The 95% CIs for sensitivity and specificity were obtained with 2000 stratified bootstrap replicates. Statistical computing was performed in R version 4.2.3, specifically with the *pROC* (ver. 1.18.2) package to perform ROC curve analyses [41].

## Results

The CFF-PR cohort consists of patients with CF (*n* = 26,392) aged 6 years of age or older monitored between January 3, 2003, and December 31, 2017. The cohort was 47.9% female (*n* = 12,634) with a median baseline age of 11.1 years (25^th^ percentile: 6.2, 75^th^ percentile: 18.9). Each study participant was followed for a median of 7.8 years (25^th^ percentile: 3.7, 75^th^ percentile: 12.4). Overall, 92.8% (*n* = 24,490) of patients self-identified as White, and 4.4% (*n* = 1172) self-identified as Black. Furthermore, 88.6% (*n* = 23,392) self-identified as non-Hispanic, and 7.7% (*n* = 2045) self-identified as Hispanic. 2.6% (*n* = 691) of White patients, 4.9% (*n* = 58) of Black patients, and 28.2% (*n* = 206) of patients who self-reported as another race did not self-report their ethnicity. Since the rate of missing reported ethnicity differs by race, we did not consider those who did not report their ethnicity. Different genetic mutations for CF were considered: 47.3% (*n* = 12,484) were F508del homozygous, 36.9% (*n* = 9744) were F508del heterozygous, and the remaining 15.8% (*n* = 4164) were neither F508del or had an unknown mutation.

**Figure 1.**
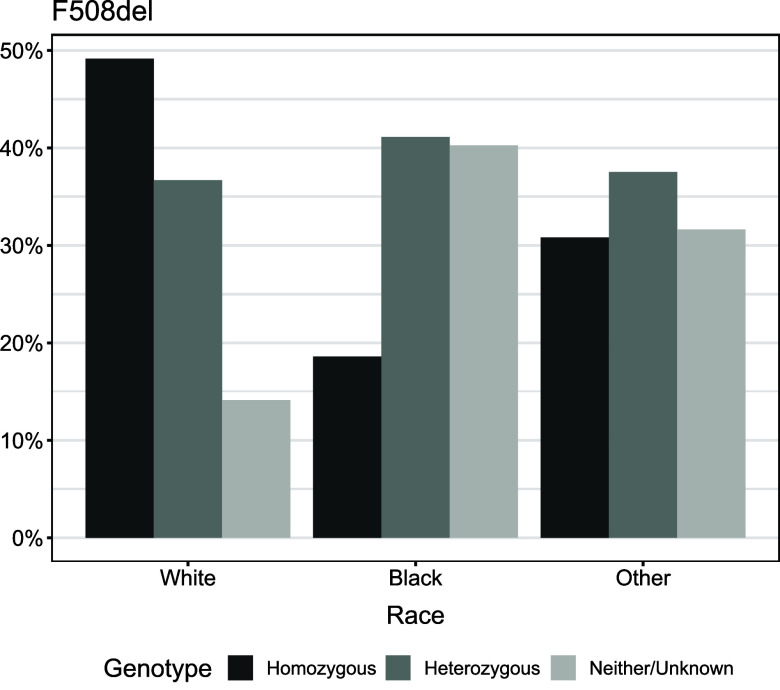
F508del mutation by racial group in the US Cystic Fibrosis Foundation Patient Registry analysis cohort.

We examined predictors and the outcome to identify possible reasons for racial discriminatory performance in the PEx precision medicine algorithm. Newborn screening for CF has rapidly expanded through DNA tests for CF mutations, so seeing differences by mutation and racial identification is not necessarily novel [42]. From Figure 1, 49.2% (*n* = 12,041) and 36.7% (*n* = 8,988) of White patients were predominately F508del homozygous or heterozygous, respectively, whereas 41.1% (*n* = 482) and 40.3% (*n* = 472) of Black patients were mainly F508del heterozygous or neither, respectively. The distribution of F508del was more balanced in patients who self-identified as another race, with 30.8% homozygous (*n* = 225), 37.5% heterozygous (*n* = 274), and 31.6% (*n* = 231) neither/unknown. Differences existed by racial group in the distribution of primary road density, secondary road density, drive time (in minutes), and straight-line distance (in kilometers) to the nearest healthcare center (Table 1). On average, Black patients lived in neighborhoods with higher densities of primary and secondary roadway, with shorter distances and shorter travel times to their CF care center. We did not see any racial differences in gender (Other: 49.0% female, Black: 48.5% female, White: 47.8% female; *p* = 0.7183) or smoking status (*p* = 0.8856), but there were racial differences in smoking households (Other: 4.7%, Black: 3.9%, White: 2.5%; *p* < 0.0001) and secondhand smoke exposure (Black: 8.1%, Other: 6.4%, White: 5.7%; *p* = 0.0020). Black patients were also less likely to have private health insurance (Black: 28.2%, Other: 37.0%, White: 51.6%; *p* < 0.0001). White patients tended to live in ZCTAs with a higher average percentage of greenspace than both Black patients or patients who self-identified as another race (White: 82.8%, Other: 72.3%, Black: 70.7%; *p* < 0.0001). Black patients were more likely to live in neighborhoods that had a higher average community deprivation index (Black: 0.404, Other: 0.364, White: 0.335; *p* < 0.0001). The average number of CF encounters was different by racial group, with White patients having a higher number of clinical visits on average (White: 38.1, Black: 31.3, Other: 29.6; *p* = 0.035). Consequently, White patients had a higher average number of encounters with a PEx (White: 9.19, Black: 8.37, Other: 6.77; *p* < 0.0001) and a higher average number of encounters with no PEx (White: 21.9, Black: 17.1, Other: 16.9; *p* < 0.0001). The average number of CF encounters per year was lowest for Black patients (Other: 5.30, White: 5.25, Black: 4.95; *p* < 0.0001). We did not see racial differences in the average rate of encounters with a PEx per year (White: 1.19, Black: 1.13, Other: 1.10; *p* = 0.0154) and in the average rate of encounters with no PEx per year (Other: 2.97, White: 2.87, Black: 2.74; *p* = 0.0436). Black patients recorded fewer clinical visits per year when compared with other races.

**Table 1.** Counts and averages of each predictor with 95% confidence intervals among the racial groups in the US Cystic Fibrosis Foundation Patient Registry analysis cohort

	Black	White	Other	p-value
Sample size	1172 (4.4%)	24,490 (92.8%)	730 (2.8%)	
Gender	F: 569 (48.5%)	F: 11,707 (47.8%)	F: 358 (49.0%)	0.7183
	M: 603 (51.5%)	M: 12,783 (52.2%)	M: 372 (51.0%)	
Private health insurance	330 (28.2%)	12,647 (51.6%)	270 (37.0%)	<0.0001
Secondhand smoke	95 (8.1%)	1394 (5.7%)	47 (6.4%)	0.0020
Living in smoking household	46 (3.9%)	608 (2.5%)	34 (4.7%)	<0.0001
Primary road density (kilometer/square meter)	1.38 (1.17, 1.60)	0.99 (0.95, 1.03)	0.81 (0.62, 0.99)	0.0001
Secondary road density (kilometer/square meter)	2.35 (2.10, 2.60)	1.92 (1.87, 1.97)	1.14 (0.89, 1.40)	<0.0001
Greenspace percent of ZIP code tabulation area	70.7% (69.2, 72.2)	82.8% (82.6, 83.1)	72.3% (70.3, 74.3)	<0.0001
Community deprivation index	0.404 (0.397, 0.410)	0.335 (0.333, 0.336)	0.364 (0.355, 0.372)	<0.0001
Straight-line distance to center (kilometers)	111.4 (90.9, 131.9)	170.6 (165.0, 176.3)	155.3 (124.8, 185.8)	<0.0001
Drivetime to healthcare center (minutes)	36.2 (35.1, 37.4)	45.6 (45.4, 45.8)	41.1 (39.8, 42.5)	<0.0001
Average number of encounters	31.3 (29.7, 32.8)	38.1 (37.7, 38.5)	29.6 (27.6, 31.5)	0.035
Average number of encounters per year	4.95 (4.66, 5.24)	5.25 (5.20, 5.30)	5.30 (5.07, 5.53)	<0.0001
Average number of encounters with a pulmonary exacerbation (PEx)	8.37 (7.82, 8.92)	9.19 (9.07, 9.31)	6.77 (6.18, 7.36)	<0.0001
Average number of encounters with PEx per year	1.13 (1.07, 1.18)	1.19 (1.18, 1.20)	1.10 (1.03, 1.18)	0.0154
Average number of encounters with no PEx	17.1 (16.3, 17.9)	21.9 (21.6, 22.1)	16.9 (15.9, 18.0)	<0.0001
Average number of encounters with no PEx per year	2.74 (2.56, 2.93)	2.87 (2.85, 2.89)	2.97 (2.84, 3.09)	0.0436

**Figure 2.**
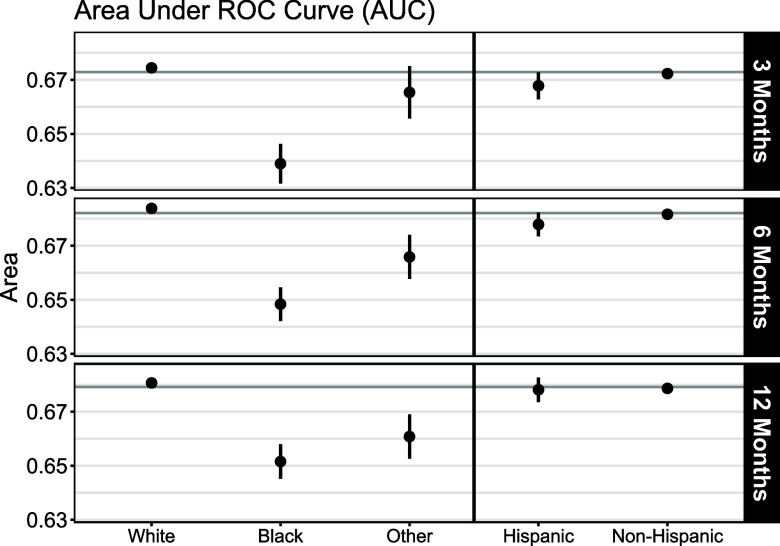
Area under the receiver operating characteristic (ROC) curve (AUC) for the 3-, 6-, and 12-month prediction horizons by racial and ethnic group. Overall AUC is indicated by the horizontal line. Group-specific AUC and their respective 95% confidence interval are displayed as points and vertical lines, respectively.

**Figure 3.**
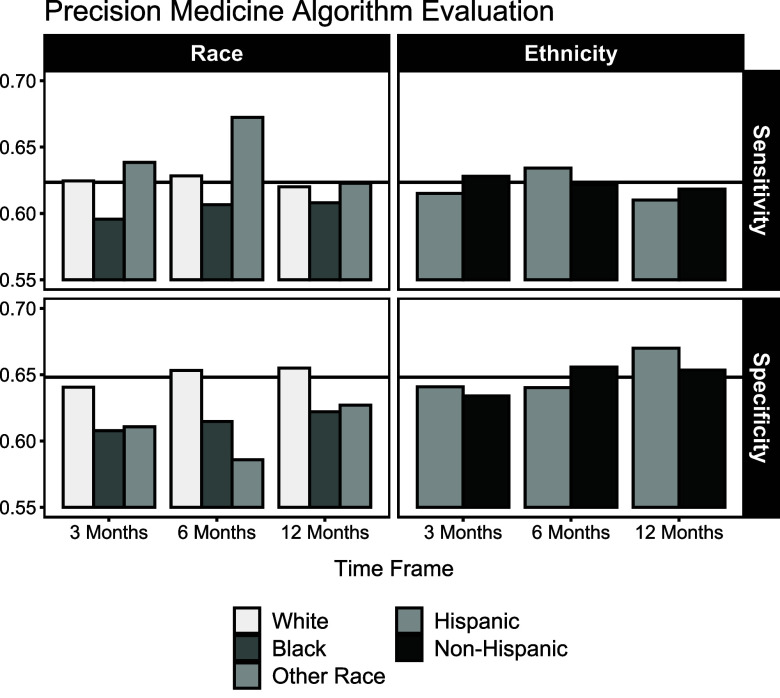
Optimal sensitivities and specificities by racial and ethnic group achieved by the precision medicine algorithm for 3-, 6-, and 12-month exacerbation prediction. The average optimal sensitivity (0.623) and average optimal specificity (0.648) are indicated by the horizontal lines.

Group-specific AUC, sensitivity, and specificity were compared with the overall AUC sensitivity and specificity to evaluate model performance between each racial and ethnic group (Figs. 2 and 3 and Supplemental Table S1). Black patients had lower sensitivity (3-month: 0.596, 95% CI: 0.582, 0.608; 6-month: 0.607, 95% CI: 0.595, 0.618; 12-month: 0.608, 95% CI: 0.598, 0.619) for every prediction horizon compared with White patients (3-month: 0.627, 95% CI: 0.625, 0.630; 6-month: 0.628, 95% CI: 0.626, 0.630; 12-month: 0.620, 95% CI: 0.618, 0.622) and those who self-identified with another racial group (3-month: 0.638, 95% CI: 0.620, 0.656; 6-month: 0.672, 95% CI: 0.657, 0.686; 12-month: 0.623, 95% CI: 0.611, 0.636). PEx predictions for Black patients also had lower specificity (3-month: 0.608, 95% CI: 0.595, 0.622; 6-month: 0.615, 95% CI: 0.604, 0.625; 12-month: 0.622, 95% CI: 0.610, 0.635) for every prediction horizon compared with White patients (3-month: 0.641, 95% CI: 0.638, 0.643; 6-month: 0.653, 95% CI: 0.651, 0.656; 12-month: 0.655, 95% CI: 0.653, 0.657) and patients who self-identified with another race (3-month: 0.611, 95% CI: 0.594, 0.626; 6-month: 0.586, 95% CI: 0.572, 0.602; 12-month: 0.627, 95% CI: 0.610, 0.643). In each case, Black patients had the worst prediction performance from the PEx precision medicine algorithm in terms of AUC, sensitivity, and specificity. Actual PEx outcomes were determined using future clinical evaluations during the prediction horizon, but the results were also similar when actual PEx outcomes were instead defined only on the date of clinical evaluation during the prediction horizon (see Supplemental Table S2).

Prior research work has shown disparities in pulmonary function exist between Hispanic and non-Hispanic patients with CF [43]. Even though non-Hispanic patients represent 88.6% of the cohort, the PEx precision medicine algorithm had similar performance between Hispanic and non-Hispanic ethnic groups. AUC values are similar for Hispanic patients (3-month: 0.668, 95% CI: 0.663, 0.673; 6-month: 0.678, 95% CI: 0.674, 0.682; 12-month: 0.678, 95% CI: 0.674, 0.683) and non-Hispanic patients (3-month: 0.672, 95% CI: 0.671, 0.674; 6-month: 0.682, 95% CI: 0.680, 0.683; 12-month: 0.679, 95% CI: 0.677, 0.680). We also allowed group-specific optimal cutoffs and verified if changing the overall optimal cutoff improves model prediction accuracy. When allowing each group to have their own optimal cutoff, we saw a similar performance from the PEx precision medicine algorithm for both racial and ethnic groups (see Supplemental Table S3). Ultimately, we found no evidence of ethnic discrimination in model prediction performance from the PEx precision medicine algorithm.

## Discussion

We characterized the accuracies of a precision medicine tool for PEx prediction by race and ethnicity, which demonstrates the need to optimize an algorithm by balancing both accuracy and group fairness. Our results show that racial, but not ethnic, differences in the PEx prediction algorithm accuracies exist when applied to the CFF-PR data. We conclude the PEx precision medicine algorithm is racially biased against Black patients with worse PEx predictions than those who self-identify with another race. These discrepancies are not due to the differences in sample size but rather by ignoring group-level fairness in prediction accuracies by racial group. Even though we see differences in model accuracies between Hispanic and non-Hispanic, groups of proportionately different sample sizes, the nature of the difference in model accuracy does not lend itself an unfair advantage to either group, since Hispanic had better sensitivity while non-Hispanic had better specificity.

The same cannot be said for the discrepancy we see between the races, and we are left to wonder why the PEx prediction algorithm yields worse sensitivity and specificity to Black patients. The PEx prediction algorithm is formulated by treating each CF individual in the CFF-PR cohort equally, so the discriminatory performance of this algorithm must be caused by some underlying factors. We examined predictors and outcome variables to formulate three main reasons for discriminatory model performance in the PEx prediction algorithm: (i) CF mutation: While the prediction algorithm is treated on individuals with CF, differences in F508del mutation exist in the cohort. The severity of CF disease and the frequency of PEx events change, in part, based on the F508del mutation [2,29], which varies between the races. (ii) Location: Black patients tended to live in locations with higher road densities. Increased roads in these areas usually lead to increased vehicle traffic and therefore are associated with increased air pollution exposure. (iii) Accessibility: Even though Black patients tended to live closer to their nearest healthcare center on average, and the drive time to arrive at their healthcare center is also lower on average, the encounter rates are noticeably lower for Black patients.

There are several potential reasons for the discrepancy – lack of interaction or trust in the healthcare system, socioeconomic status (SES), and affordability for essential healthcare services, accessibility, and the quality of health care, all of which have been studied more generally in environmental health research [44,45]. More frequent clinic visits are associated with better lung function outcomes in CF [13], but there has been a shift in the care paradigm toward telehealth visits that were partially motivated to overcome barriers to access that were heightened during the coronavirus disease 2019 pandemic [46]. However, recent research on telehealth utilization in US CF patients during the pandemic showed that individuals who identified as Black or Hispanic/Latino and those who reported having financial constraints were less likely to have a telehealth visit [46]. Although individual-level proxies of SES (e.g., Medicaid/state insurance use) are linked to lung function and survival in CF, Albon and colleagues found no association between SES factors and telehealth utilization in the aforementioned study, and differences due to race/ethnicity persisted after SES adjustments. Coupling prior literature with our study findings, interventions to improve chronic disease management, including outcome prognostication, for Black people with CF may have suboptimal impact unless fairness is considered.

The obstacles to algorithm fairness that we identified also pose challenges for therapeutics development, which are expected to grow in light of the changing demographics of CF worldwide [47,48]. While there is generally a paucity of transparent (i.e., freely open) algorithms that are subjected to critical appraisal from and co-production by patient communities [49], the CF patient subgroups identified from our study at greatest risk for algorithmic bias have also historically been underrepresented in CF clinical research (e.g., identifying as nonwhite, rural, or socioeconomically disadvantaged) [50,51]. Although research participation among CF minority populations has been a longstanding challenge in CF, it is of greater importance in the modern era of care, given the need to develop therapies for the remaining 5%–6% of the US CF population for whom highly effective modulator therapies are currently unavailable [52]. Individuals who have ultra-rare mutations that are not FDA approved for modulator treatment tend to identify as Black/African American or nonwhite Hispanic, and they have the lower average lung function, compared with their white counterparts [53].

A larger issue that is raised from this research is how to address group-level fairness in CF precision medicine tools. Although unintentional, both (i) associations of model predictors with race and (ii) associations of our proxies for health outcomes with race can lead to prediction algorithms that could replicate systemic racism in the data and the system used to create it. Clinical implementation of this PEx prediction tool could possibly enhance racial disparities in access to care for CF patients. Individual-level fairness and group-level fairness cannot be maximized simultaneously in a prediction algorithm [28]. Therefore, we must examine the tradeoff in the accuracy of the PEx prediction algorithm with respect to group-level fairness [14]. Addressing group-level fairness in precision medicine models, particularly in respiratory diseases like CF, is essential to ensure that these tools benefit all patients equitably, irrespective of their race, ethnicity, or socioeconomic background. There have been some methods suggested to effectively address bias and factors to consider for inclusion or exclusion in these models. These suggestions include (i) diverse data representation which ensures that the data used to train precision medicine models are representative of the diverse patient population affected by the condition [22], (ii) bias detection and correction which may require using advanced statistical tools to detect and correct biases in the model [54], and (iii) group-specific model adjustment that allows developing separate models for groups with distinct characteristics or adjusting models to account for known disparities [21].

There is a critical lack of evaluation of racial and ethnic fairness in precision health medicine within CF patients. Discrepancies of the PEx prediction algorithm on the CFF-PR cohort by racial and ethnic group must raise the awareness of group-level bias in precision medicine algorithm development in CF research. We hope to invite discussions on how to promote ways of addressing group-level fairness in statistical modeling research. By not addressing group fairness, researchers run the risk of developing statistical models that puts those from minority populations at a strong disadvantage when it comes to model accuracy and performance, further exacerbating racial inequalities in CF outcomes and care. Precision medicine tools can then be developed to better meet the needs of healthcare professionals and promote equitable patient care.

## Supporting information

Colegate et al. supplementary material Colegate et al. supplementary material

## Supplementary material

The supplementary material for this article can be found at https://doi.org/10.1017/cts.2024.532.
