# Supplementary material for: Evaluating precision medicine tools in cystic fibrosis for racial and ethnic fairness
Source: J Clin Transl Sci. 2024 May 7;8(1):e94. doi: 10.1017/cts.2024.532 (PMC11362628; doi:10.1017/cts.2024.532)
Supplement: Colegate et al. supplementary material [file S2059866124005326sup001.docx]

**Supplement**

ROC curves are formulated by comparing PEx probability from the precision medicine algorithm to actual PEx outcomes. Table S1 shows the group-specific AUC, sensitivity, and specificity compared to the overall AUC sensitivity, and specificity. There is some discrepancy as to how to determine whether a FIES-defined PEx will occur within the prediction horizon at a clinical evaluation. To determine actual PEx outcomes, we examined all future patient encounters from the date of the clinical evaluation to the end of the prediction horizon. We classified a patient to have experienced a PEx during the prediction horizon when they were clinically evaluated and confirmed to have met the FIES-criteria within the prediction horizon at least once. There are possibly better methods to determine actual PEx outcomes than simply monitoring each patient during future clinical visits.

Using future clinical evaluations to determine actual PEx outcomes negatively impacts the accuracy of predictions made by the precision medicine algorithm. We evaluated predictions made by the PEx precision medicine algorithm to see whether a FIES-defined PEx was confirmed to be present on the date of a clinical evaluation. We formulated ROC curves by contrasting a patient’s PEx probability from the precision medicine algorithm to actual PEx outcomes only on the date of the clinical evaluation. Group-specific ROC curves were implemented using the overall optimal cutoff probability to determine group-specific sensitivity and specificity. Table S2 shows the AUC, sensitivity, and specificity of the overall and group-specific ROC curves when evaluating for a PEx during a clinical visit. The overall AUC to evaluate the precision medicine algorithm is noticeably higher for every prediction horizon when using the date of clinical evaluation (3-month: 0.731, 95% CI: (0.729, 0.732); 6-month: 0.739, 95% CI: (0.738, 0.740); 12-month: 0.752, 95% CI: (0.751, 0.753)) compared to screening clinical evaluations for a PEx within the prediction horizon (3-month: 0.673, 95% CI: (0.672, 0.674); 6-month: 0.682, 95% CI: (0.681, 0.683); 12-month: 0.679, 95% CI: (0.678, 0.680)). The decrease in the accuracy of the precision medicine algorithm is not necessarily due to the probabilities being calculated but rather how the PEx outcome is determined. Future work will examine how to best determine the actual PEx outcome, whether through masking, modeling future FEV_1_ trajectories, or via other approaches.

Since the CFF-PR cohort is predominately white (92.8%) and non-Hispanic (88.6%), we expected the selection of the optimal cutoff probability to be highly influenced by these individuals. We repeated the ROC curve analysis by allowing race- and ethnic-specific optimal cutoffs. Table S3 shows the AUC, sensitivity, and specificity for each racial and ethnic group where each is allowed to have their own group-specific optimal cutoff probability. The optimal cutoff probability varies widely between White patients (3-month: 0.523, 6-month: 0.464, 12-month: 0.409), Black patients (3-month: 0.457, 6-month: 0.415, 12-month: 0.417) and patients who self-identified with another race (3-month: 0.521, 6-month: 0.488, 12-month: 0.448) at every prediction horizon. The group-specific cutoff probabilities allow the precision medicine algorithm to maximize both sensitivity and specificity for each race. This seems to alleviate some of the discrepancy in sensitivity and specificity between Black patients and White patients. Predictions made on Black patients still suffer from model accuracy. Sensitivity, for example, appears to be similar for Black patients (3-month: 0.631, 6-month: 0.635, 12-month: 0.605) when compared to White patients (3-month: 0.625, 6-month: 0.629, 12-month: 0.620) and patients of another race (3-month: 0.640, 6-month: 0.624, 12-month: 0.606). Specificity is still lowest for Black patients (3-month: 0.576, 6-month: 0.592, 12-month: 0.626) than for both White patients (3-month: 0.641, 6-month: 0.653, 12-month: 0.655) and patients of another race (3-month: 0.611, 6-month: 0.635, 12-month: 0.650). This leads us to believe that calibrating the precision medicine algorithm to ensure group-fairness leads to fairer prediction accuracies between racial groups.

|  | Overall | White | Black | Other | Hispanic | Non-Hispanic |
| --- | --- | --- | --- | --- | --- | --- |
| 3 Months | | | | | | |
| Cutoff | 0.523 | 0.523 | 0.523 | 0.523 | 0.523 | 0.523 |
| AUC + 95% CI | 0.673  (0.672, 0.674) | 0.674  (0.673, 0.676) | 0.639  (0.632, 0.646) | 0.665  (0.656, 0.675) | 0.668  (0.663, 0.673) | 0.672  (0.671, 0.674) |
| Sensitivity + 95% CI | 0.624  (0.621, 0.626) | 0.627  (0.625, 0.630) | 0.596  (0.582, 0.608) | 0.638  (0.620, 0.656) | 0.615  (0.606, 0.624) | 0.628  (0.625, 0.631) |
| Specificity + 95% CI | 0.639  (0.636, 0.641) | 0.641  (0.638, 0.643) | 0.608  (0.595, 0.622) | 0.611  (0.594, 0.626) | 0.641  (0.631, 0.650) | 0.634  (0.631, 0.637) |
| 6 Months | | | | | | |
| Cutoff | 0.465 | 0.465 | 0.465 | 0.465 | 0.465 | 0.465 |
| AUC + 95% CI | 0.682  (0.681, 0.683) | 0.684  (0.683, 0.685) | 0.648  (0.642, 0.655) | 0.666  (0.658, 0.674) | 0.678  (0.674, 0.682) | 0.682  (0.680, 0.683) |
| Sensitivity + 95% CI | 0.627  (0.625, 0.630) | 0.628  (0.626, 0.630) | 0.607  (0.595, 0.618) | 0.672  (0.657, 0.686) | 0.634  (0.626, 0.642) | 0.622  (0.620, 0.624) |
| Specificity + 95% CI | 0.652  (0.649, 0.654) | 0.653  (0.651, 0.656) | 0.615  (0.604, 0.625) | 0.586  (0.572, 0.602) | 0.640  (0.632, 0.648) | 0.656  (0.653, 0.658) |
| 12 Months | | | | | | |
| Cutoff | 0.409 | 0.409 | 0.409 | 0.409 | 0.409 | 0.409 |
| AUC + 95% CI | 0.679  (0.678, 0.680) | 0.681  (0.679, 0.682) | 0.652  (0.645, 0.658) | 0.661  (0.653, 0.669) | 0.678  (0.674, 0.683) | 0.679  (0.677, 0.680) |
| Sensitivity + 95% CI | 0.619  (0.617, 0.621) | 0.620  (0.618, 0.622) | 0.608  (0.598, 0.619) | 0.623  (0.611, 0.636) | 0.610  (0.603, 0.617) | 0.618  (0.616, 0.620) |
| Specificity + 95% CI | 0.654  (0.652, 0.656) | 0.655  (0.653, 0.657) | 0.622  (0.610, 0.635) | 0.627  (0.610, 0.643) | 0.670  (0.661, 0.679) | 0.653  (0.651, 0.656) |

**Table S1:** Optimal cutoff, area under the ROC curve (AUC) with 95% confidence interval, sensitivity, and specificity with their respective 95% confidence intervals by racial and ethnic group that was achieved by the PEx precision medicine algorithm for three-, six-, and twelve-month PEx prediction, as shown in Figures 2-3.

|  | Overall | White | Black | Other | Hispanic | Non-Hispanic |
| --- | --- | --- | --- | --- | --- | --- |
| 3 Months | | | | | | |
| Cutoff | 0.489 | 0.489 | 0.489 | 0.489 | 0.489 | 0.489 |
| AUC + 95% CI | 0.731  (0.729, 0.732) | 0.731  (0.730, 0.733) | 0.712  (0.706, 0.718) | 0.730  (0.722, 0.738) | 0.727  (0.722, 0.731) | 0.730  (0.729, 0.732) |
| Sensitivity | 0.690 | 0.690 | 0.676 | 0.717 | 0.738 | 0.685 |
| Specificity | 0.667 | 0.668 | 0.649 | 0.639 | 0.613 | 0.672 |
| 6 Months | | | | | | |
| Cutoff | 0.536 | 0.536 | 0.536 | 0.536 | 0.536 | 0.536 |
| AUC + 95% CI | 0.739  (0.738, 0.740) | 0.739  (0.738, 0.741) | 0.722  (0.716, 0.728) | 0.740  (0.731, 0.748) | 0.735  (0.730, 0.739) | 0.750  (0.737, 0.740) |
| Sensitivity | 0.696 | 0.696 | 0.685 | 0.692 | 0.739 | 0.691 |
| Specificity | 0.674 | 0.675 | 0.658 | 0.664 | 0.627 | 0.678 |
| 12 Months | | | | | | |
| Cutoff | 0.605 | 0.605 | 0.605 | 0.605 | 0.605 | 0.605 |
| AUC + 95% CI | 0.752  (0.751, 0.753) | 0.753  (0.752, 0.754) | 0.739  (0.733, 0.745) | 0.756  (0.748, 0.764) | 0.749  (0.745, 0.753) | 0.752  (0.751, 0.753) |
| Sensitivity | 0.696 | 0.697 | 0.685 | 0.712 | 0.739 | 0.692 |
| Specificity | 0.693 | 0.693 | 0.685 | 0.684 | 0.653 | 0.696 |

**Table S2:** Area under the Receiver Operating Characteristic (ROC) curve (AUC) with 95% confidence interval (CI), optimal sensitivity and specificity overall by racial and ethnic group achieved by the precision medicine algorithm when actual exacerbation (PEx) outcomes are defined only on the date of clinical evaluation during the three-, six-, and twelve-month prediction horizon.

|  | Overall | White | Black | Other | Hispanic | Non-Hispanic |
| --- | --- | --- | --- | --- | --- | --- |
| 3 Months | | | | | | |
| Cutoff | 0.523 | 0.523 | 0.457 | 0.521 | 0.642 | 0.503 |
| AUC + 95% CI | 0.673  (0.672, 0.674) | 0.674  (0.673, 0.676) | 0.639  (0.632, 0.646) | 0.665  (0.656, 0.675) | 0.668  (0.663, 0.673) | 0.672  (0.671, 0.674) |
| Sensitivity | 0.624 | 0.625 | 0.631 | 0.640 | 0.615 | 0.628 |
| Specificity | 0.639 | 0.641 | 0.576 | 0.611 | 0.641 | 0.634 |
| 6 Months | | | | | | |
| Cutoff | 0.465 | 0.464 | 0.415 | 0.488 | 0.534 | 0.467 |
| AUC + 95% CI | 0.682  (0.681, 0.683) | 0.684  (0.683, 0.685) | 0.648  (0.642, 0.655) | 0.666  (0.658, 0.674) | 0.678  (0.674, 0.682) | 0.682  (0.680, 0.683) |
| Sensitivity | 0.627 | 0.629 | 0.635 | 0.624 | 0.634 | 0.622 |
| Specificity | 0.652 | 0.653 | 0.592 | 0.635 | 0.640 | 0.656 |
| 12 Months | | | | | | |
| Cutoff | 0.409 | 0.409 | 0.417 | 0.448 | 0.516 | 0.402 |
| AUC + 95% CI | 0.679  (0.678, 0.680) | 0.681  (0.679, 0.682) | 0.652  (0.645, 0.658) | 0.661  (0.653, 0.669) | 0.678  (0.674, 0.683) | 0.679  (0.677, 0.680) |
| Sensitivity | 0.619 | 0.620 | 0.605 | 0.606 | 0.610 | 0.618 |
| Specificity | 0.654 | 0.655 | 0.626 | 0.650 | 0.670 | 0.653 |

**Table S3:** Area under the Receiver Operating Characteristic (ROC) curve (AUC) with 95% confidence interval (CI), optimal sensitivity and specificity overall by racial and ethnic group achieved by the precision medicine algorithm for group-specific optimal cutoff probabilities.
